# Biosensing with Silicon Nitride Microring Resonators Integrated with an On-Chip Filter Bank Spectrometer

**DOI:** 10.1021/acssensors.2c02276

**Published:** 2023-02-14

**Authors:** Michael R. Bryan, Jordan N. Butt, Joseph Bucukovski, Benjamin L. Miller

**Affiliations:** † Department of Dermatology, University of Rochester, Rochester, New York 14627, United States; ‡ Department of Chemistry, University of Rochester, Rochester, New York 14627, United States; § Department of Biochemistry and Biophysics, University of Rochester, Rochester, New York 14627, United States; ∥ Institute of Optics, University of Rochester, Rochester, New York 14627, United States

**Keywords:** microring resonators, photonic sensors, filter bank spectrometers, biosensing, AIM Photonics

## Abstract

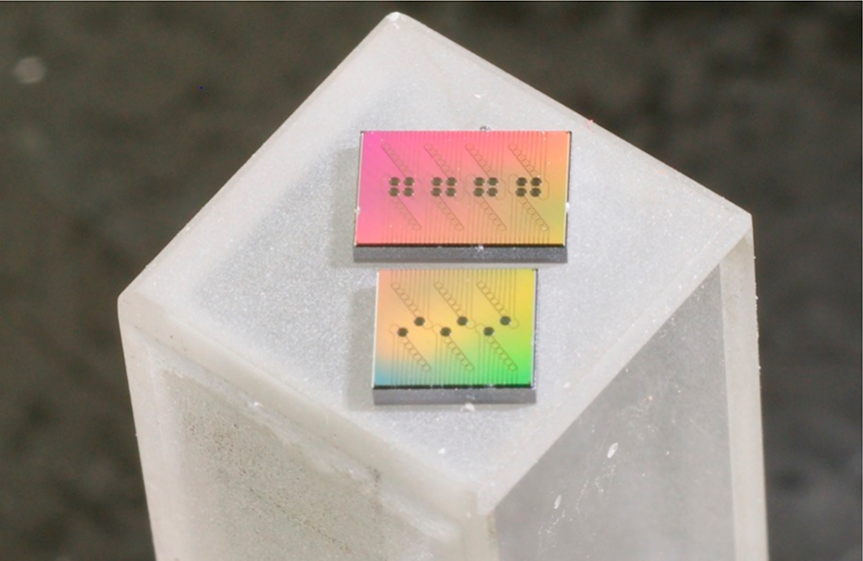

Wearable, mobile, and point-of-care (POC) sensors comprise a rapidly expanding field of devices aimed at improving human health by relaying real-time biometric data such as heart rate and glucose levels. The current scope of what these devices can offer healthcare is limited by their inability to measure biomarkers associated with inflammation, well-being, and disease. Photonic biosensors that integrate sensing elements directly with spectrometers, lasers, and detectors are an attractive approach to enabling POC sensors, with distinct advantages in terms of size, weight, power consumption, and cost. Here, we have demonstrated for the first time the integration of photonic microring resonator biosensors with an on-chip microring filter bank spectrometer for the controlled detection of inflammatory biomarker C-reactive protein (CRP) in serum. We demonstrate that sensor and spectrometer performance is tolerant of temperature variation, as temperature dependence moves in parallel. Finally, we assess the impact of manufacturing variability on the 300 mm wafer scale on the performance of the spectrometer. Taken together, these results suggest that integration of on-chip ring filter bank spectrometers with ring resonator-based biosensors constitutes an attractive approach toward cost-effective integrated sensor development.

Laboratory diagnostics have revolutionized modern medicine by improving the diagnosis, prognosis, and treatment of disease. However, state-of-the-art laboratory diagnostics are not suitable for wearable, mobile, or point-of-care (POC) applications because they are large, require skilled operators, and are costly to acquire and maintain. There is substantial interest in developing alternative diagnostic platforms that obviate the limitations of current technologies.^1,2^ Integrated photonic approaches to biosensing offer clear advantages in size, weight, power, and cost by leveraging the established semiconductor manufacturing infrastructure. Examples of photonic structures used for sensing include ring resonators,^3,4^ photonic crystals,^5−7^ and Mach–Zehnder interferometers.^8−10^

When a device is fully reusable, monolithic integration of components (such as a laser source and detector) decreases size and cost. For biosensing applications, it is desirable to make certain components, such as the signal transducer, single-use and disposable. An open question is whether there is a middle approach, where a transducer and spectrometer may be integrated without significantly increasing cost.

Several approaches to integrated photonic spectrometry have been demonstrated. Miniaturized dispersive systems such as arrayed waveguide gratings (AWGs)^11^ and Echelle gratings^12,13^ have been used to scale down traditional free-space optical spectrometer systems. Biosensing with these devices has typically focused on detection of analytes by their Raman or infrared spectral signature.^14^ While there are examples of sensors integrated with AWGs for biosensing,^15,16^ one of the challenges with this configuration of the transducer and spectrometer is the disparity between the temperature dependence of the ring resonator and the AWG. Here, we show that integrating ring resonators for sensing with ring filter banks for spectrometry alleviate this concern over divergent temperature dependence.

Spectrometers have been used in a range of applications including spectroscopy to study the spectral features of atoms, molecules, and bulk materials. Here, an integrated filter bank spectrometer is used to monitor the spectral characteristics of an upstream ring resonator that is exposed to the environment for sensing.

In general, spectrometers are optimized for high resolution by increasing channel count and density while decreasing simultaneous activation of adjacent channels. This is critical for identifying the narrow spectral features of molecular Raman signals. However, when the spectral feature is comparatively broad, such as the drop spectrum of an add/drop ring resonator, the resonance wavelength may be identified using fewer, sparsely placed channels (Figure 1). Narrowband filters such as microring filter banks^17,18^ have been used extensively for spectral analysis and signal processing but, as far as we are aware, have not been integrated with transducers in a biosensing context.

**Figure 1:**
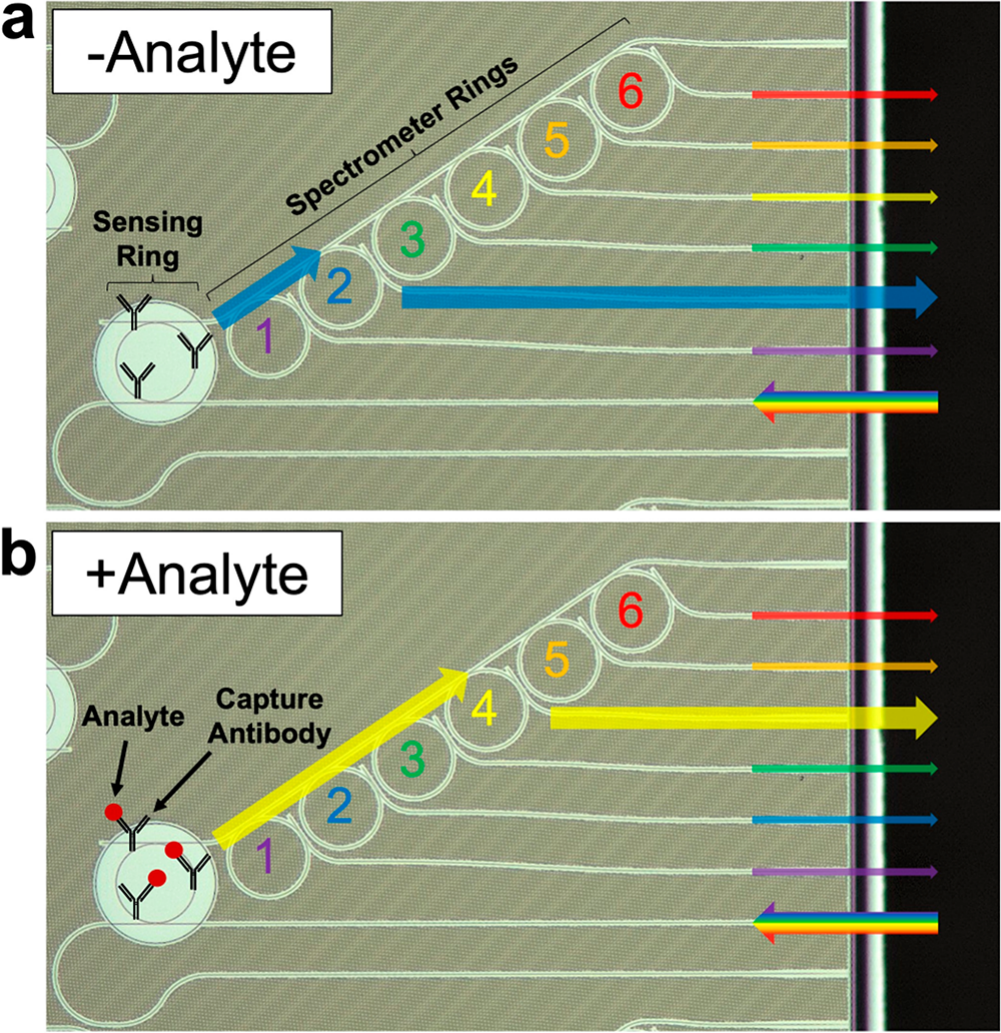
Working principle of sensing ring resonator integrated with ring filter bank for spectrometry. The exposed sensing ring may have covalently linked capture antibodies on the surface. A tunable laser source is aligned to the input of the sensing ring, and the wavelength is scanned. (a) In the absence of an analyte, the resonance wavelength of the sensing ring corresponds to the resonance wavelength of the second spectrometer ring, and the greatest power is measured from channel 2. (b) When the analyte is bound to the capture antibody, the resonance wavelength of the sensing ring shifts toward longer wavelengths. This wavelength is now closer to that of spectrometer ring 4, and the greatest power is measured from channel 4.

In this paper, we demonstrate this concept for bulk sensing of sucrose solutions and specific detection of C-reactive protein (CRP) in serum at biologically relevant concentrations. To the best of our knowledge, this is the first reported example of such a device and sets the stage for further development of spectrometer-integrated photonic biosensors.

## Experimental Techniques

### Materials

The C-reactive protein (CRP) and anti-CRP antibody were a gift from Ortho Clinical Diagnostics (Rochester, NY). Antifluorescein (anti-FITC) and fetal bovine serum (FBS) were obtained from Rockland Immunochemicals (Limerick, PA). CRP was diluted in 20% FBS in assay wash buffer (AWB), by volume. AWB is composed of modified phosphate-buffered saline–potassium-free (mPBS), 3 mM EDTA, and 0.01% Tween-20.

### Microring Resonators

Modeling of silicon nitride ring waveguides was performed using the finite difference (FD) method in Synopsys OptoDesigner. Silicon nitride waveguides were modeled as 1.5-μm-wide and 220-nm-thick with a refractive index of 1.98, silicon dioxide cladding with refractive index 1.44, and silicon substrate with refractive index of 3.48. Sensing ring waveguides were modeled with an upper water cladding of refractive index 1.32. Once the effective index of the guided mode, bending loss, and substrate loss had been determined by FD, coupled mode theory (CMT) in Synopsys OptoDesigner was used to determine the optimum gap between the bus waveguide and ring resonator. Coupling gaps were designed to achieve broad low-Q sensing ring resonance and narrower high-Q spectrometer ring resonance.

**Figure 2:**
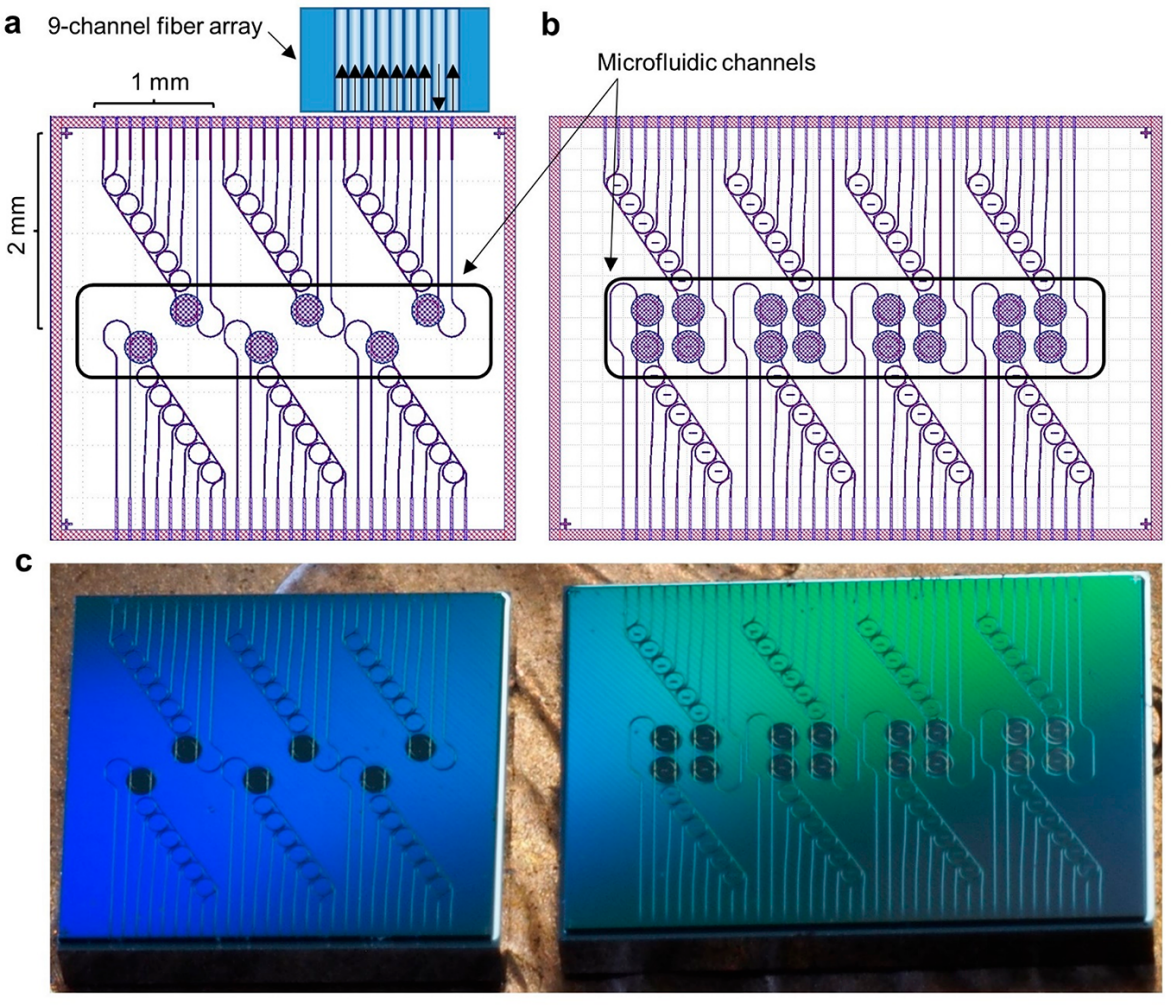
(a) A 4 × 4.4 mm layout containing six sensor ring-coupled filter banks. These devices, used for bulk sensing experiments, each have a footprint of 2 × 1 mm, a single input, a transmitted output from the sensing ring T_1_, six filter bank outputs, and a transmitted output from the filter bank bus waveguide T_2_. Nine edge coupled waveguides are addressed simultaneously by a nine-channel fiber array for optical input and output. The microfluidic channel outlined in black enables fluid delivery to the sensing rings. (b) A 4 × 5.8 mm layout containing eight dual-ring-coupled filter banks used for controlled biosensing experiments. (c) Diced filter bank die as received from the vendor.

Several sensing ring-coupled spectrometer devices were incorporated onto a larger chip, as shown in Figure 2a, for ease of handling and testing of several designs during a single experiment. A single device or many could be used per chip for a typical use case (i.e., in diagnostics or in a field-deployable biosensor) to either minimize the size and cost or facilitate multiplexing and statistical averaging. Two types of sensing ring-coupled spectrometer devices were designed. The first type, shown in Figure 2a, contained a single add/drop sensing ring upstream of six spectrometer rings. The spectrometer rings share a single bus waveguide and have slightly different circumferences, leading to different resonant wavelengths. This type of device was used for bulk sensing experiments, where the sensing ring resonance changes in response to changing bulk refractive index of the solution above the ring. However, for targeted detection of an analyte, it is desirable to have a second ring to serve as a negative control and enable correction for nonspecific binding and shift due to change in the background refractive index. To accomplish specific biosensing, we designed a device with two sensing rings upstream of the filter bank (Figure 2b). In this case, one ring serves as a negative control while the other is functionalized with capture antibodies specific to the analyte of interest.

Exposed sensing rings were designed with a diameter of 197.270 μm and a coupling gap of 650 nm to yield an FSR of ∼2.2 nm at a wavelength of 1550 nm under aqueous cladding. Oxide-clad spectrometer rings were designed with a diameter of 197.169 μm and a coupling gap of 750 nm to achieve an identical FSR range of ∼2.2 nm.

### Photonic Chip Fabrication

The photonic sensor/spectrometers were fabricated via photolithography using a 300 mm wafer process available at AIM Photonics in Albany, New York.^19^ Fabricated wafers were diced and returned on dicing tape. An image of a diced filter bank die, as received from the dicing vendor, is shown in Figure 2c. A complete description of the AIM Photonics passives MPW format from which our layer stack derives has been reported previously.^19^

The measured Q-factor of fabricated sensing (aqueous-clad) and spectrometer (oxide-clad) rings was 1.3 × 10^4^ and 2.4 × 10^5^, respectively. As fabricated, the FSR of sensing and spectrometer rings differed by 60 pm, measured as 2.183 and 2.123 nm, respectively.

### Photonic Sensor Chip Functionalization

Diced sensor chips were removed from the dicing tape and washed for 15 min in a 1:1 mixture of hydrochloric acid and methanol, then rinsed sequentially in four Petri dishes filled with Nanopure water. Another wash was performed for 15 min in a 3:1 mixture of sulfuric acid and 25% hydrogen peroxide (“piranha” solution; ****Caution!***Piranha solution is highly caustic and reacts violently with organics*), rinsed sequentially with Nanopure water in four Petri dishes, and finally dried with nitrogen. Next, the chips were submerged in a solution of 1% (3-triethoxysilyl)propylsuccinic anhydride (Gelest, Morrisville, PA) in anhydrous toluene (freshly distilled over sodium metal under a nitrogen atmosphere) for 40 min and subsequently rinsed in anhydrous toluene for 1 min and dried with nitrogen. The chips were transferred to a 110 °C oven for 30 min to cure the silane layer and then used immediately.

Antibodies were covalently attached to the functionalized chip surface by printing antibody solution directly on the sensing rings using a Scienion SciFLEXARRAYER SX microarrayer (Figure S1b). The probe rings were spotted with approximately 3 nL of anti-CRP at 400 μg/mL in mPBS (pH 7.2), and the control rings received anti-FITC at 600 μg/mL in mPBS (pH 5.8; Figure S1c). These were incubated in the microarrayer at 75% humidity for 30 min before overspotting an identical volume of StabilCoat Plus (Surmodics; Figure S1d). After a subsequent 30 min, the chips were removed from the 75%-humidity arrayer chamber and placed into a small desiccator with fresh desiccant at room temperature for at least 4 h, as the manufacturer suggests. In our hands, coated chips have a shelf life of at least several months.

### Microfluidic Assembly

**Figure 3:**
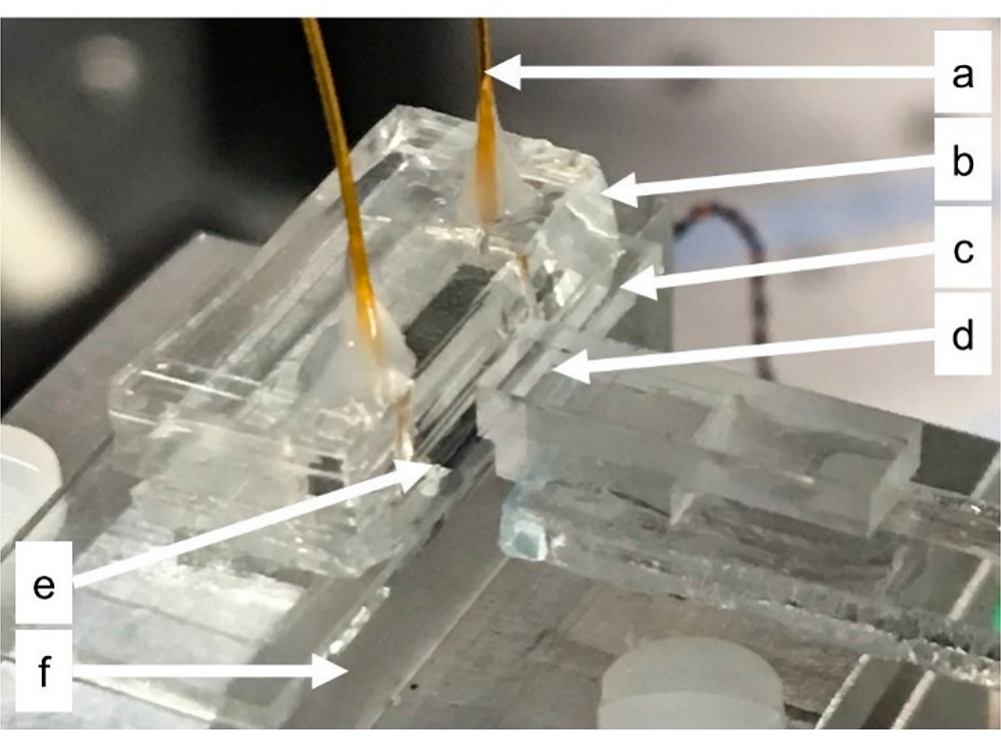
(a) Polyimide tubing for fluidic input and output. The input side connects to the pressure-driven pump, and the output drains to a receptacle. (b) PDMS gasket with two narrow vertical openings for polyimide tubing. (c) Two layers of 3M adhesive channels. (d) Fiber array optical input and output. (e) Photonic chip with edge exposed to allow optical coupling. (f) Temperature controlled stage.

To enable efficient sample delivery to the sensor surface, the device was packaged into a microfluidic assembly (Figure 3). The sensor was surrounded on three sides with silicon substrates to provide a uniform surface that extended several millimeters beyond the chip perimeter. Next, 3M adhesive transfer tape (467MP) was cut with a Silhouette Cameo craft cutter to define a boundary around the exposed sensing rings and to form a sealing layer. On top of that, a second layer of 3M adhesive was cut to define a channel connecting input and output fluidic ports beyond the boundary of the sensor. Finally, a PDMS gasket with polyimide tubing for input and output was aligned to the adhesive channel.

### Measurement Apparatus

The measurement apparatus is presented in Figure S2. A tunable laser source was aligned to the PIC microfluidic assembly by maximizing coupling between the waveguides of the PIC and a fiber array with nine single mode SMF-28 fibers on a 127-μm pitch (OZ Optics). Light from the tunable laser source (Keysight N7745A) was routed through a polarization controller (Thorlabs FPC561) to ensure linearly polarized light was properly oriented for the TE mode of the waveguide. Output light captured by fibers of the same fiber array was routed to each of the eight channels of the optical power meter (Keysight N7745A). The tunable laser and optical power meter were connected to a computer via a GPIB interface and controlled using Keysight’s Photonic Application Suite. Optical alignment was assisted by a dual camera VIS/IR microscope consisting of a 5× IR objective lens (Mitutoyo Plan Apo NIR 46-402) fitted to a 6.5× zoom lens with coaxial illumination (Thorlabs MVL6X3Z) and a dual-camera mount (Thorlabs 2SCM1-DC) with a long-pass dichroic mirror (Thorlabs DMLP950R) to pass IR light to the IR camera (WiDy InGaAs 650) and reflect visible light to the CMOS camera (Thorlabs DCC1645C).

### Spectral Measurements and Sample Addition

**Figure 4:**
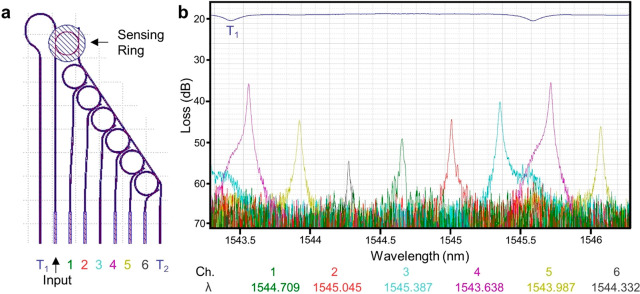
(a) From left to right, the edge-coupled waveguides of a sensing ring-coupled filter bank are the transmitted output from the sensing rings bus T_1_, the input, the six outputs from the filter bank spectrometer rings, and the transmitted output from the filter bank bus T_2_. (b) Example spectra from a sensing ring-coupled filter bank. The transmitted output T_1_ carries the resonance notch from the sensing ring. Likewise, the transmitted output from the filter bank bus waveguide T_2_ is composed of the sensing resonance peak and the resonance notch for each spectrometer ring (not shown). The signal coupled to the filter bank bus waveguide must pass each of the spectrometer rings 1 through 6 in sequence. When the wavelength of light in the filter bank bus waveguide is identical to the resonant wavelength of a filter bank ring, the resonance notch is observed in the transmitted signal T_2_ and a corresponding peak is observed in the output for that spectrometer channel. The height of the spectrometer channel peak is inversely related to the difference between the resonance wavelengths of the spectrometer and sensing ring.

Once the device was packaged in the microfluidic assembly and aligned, 80 nm spectra were recorded continuously at 1 pm resolution from 1500 to 1580 nm. Each scan took about 8 s to complete. All 8 channels of the optical power meter were monitored simultaneously using the insertion loss interface of the Keysight Photonic Application suite. Example spectra are shown in Figure 4.

Samples were introduced to the sensor surface using a Fluigent EZ system with M-switch (North Chelmsford, MA) at a flow rate of 15 μL/min. For bulk sensing experiments to determine the refractive index sensitivity of the devices, sucrose solutions with concentrations ranging from 0 to 10% (v/v) were sequentially introduced, with each solution allowed to flow until the resonance stabilized, and the spectra recorded. For biosensing experiments, the 20% FBS/AWB solution was pumped over the surface for 15 min to remove the StabilCoat Plus from sensing rings and block the ancillary surfaces of the sensor and microfluidic channel. Subsequently, increasing concentrations of CRP diluted in 20% FBS/AWB were pumped through the sensor for 15 min at a time, and the spectra wererecorded. The observed resonance wavelengths of the sensing rings generally stabilized after ∼10 min.

### Analysis of Spectra

Collected spectra were processed automatically through a custom Python script, modified from that which was described previously.^3^ The output spectra for each channel were collected simultaneously and stored in the same file. Minor modifications were made to the pipeline to accommodate multiple output channels. Briefly, spectral features including peak location, peak height, quality factor, chi-squared values, and peak fitting parameters are extracted by fitting the peak features with Lorentzian functions. As shown in Figure 4b, the transmitted T_1_ signal alone was sufficient to determine the resonance wavelength of the sensing ring. However, correlating the transmitted power with the tunable laser wavelength during a scan requires synchronizing the laser and detectors, adding expense and complexity to the apparatus. This requirement was obviated by instead, recording the maximum power transmitted through each of the spectrometer channels during a scan. Then, the maximum power and wavelength of the spectrometer channels were used to determine the resonance wavelength of the sensing ring.

## Experimental Results

### Bulk Sensing Sucrose Solutions

**Figure 5:**
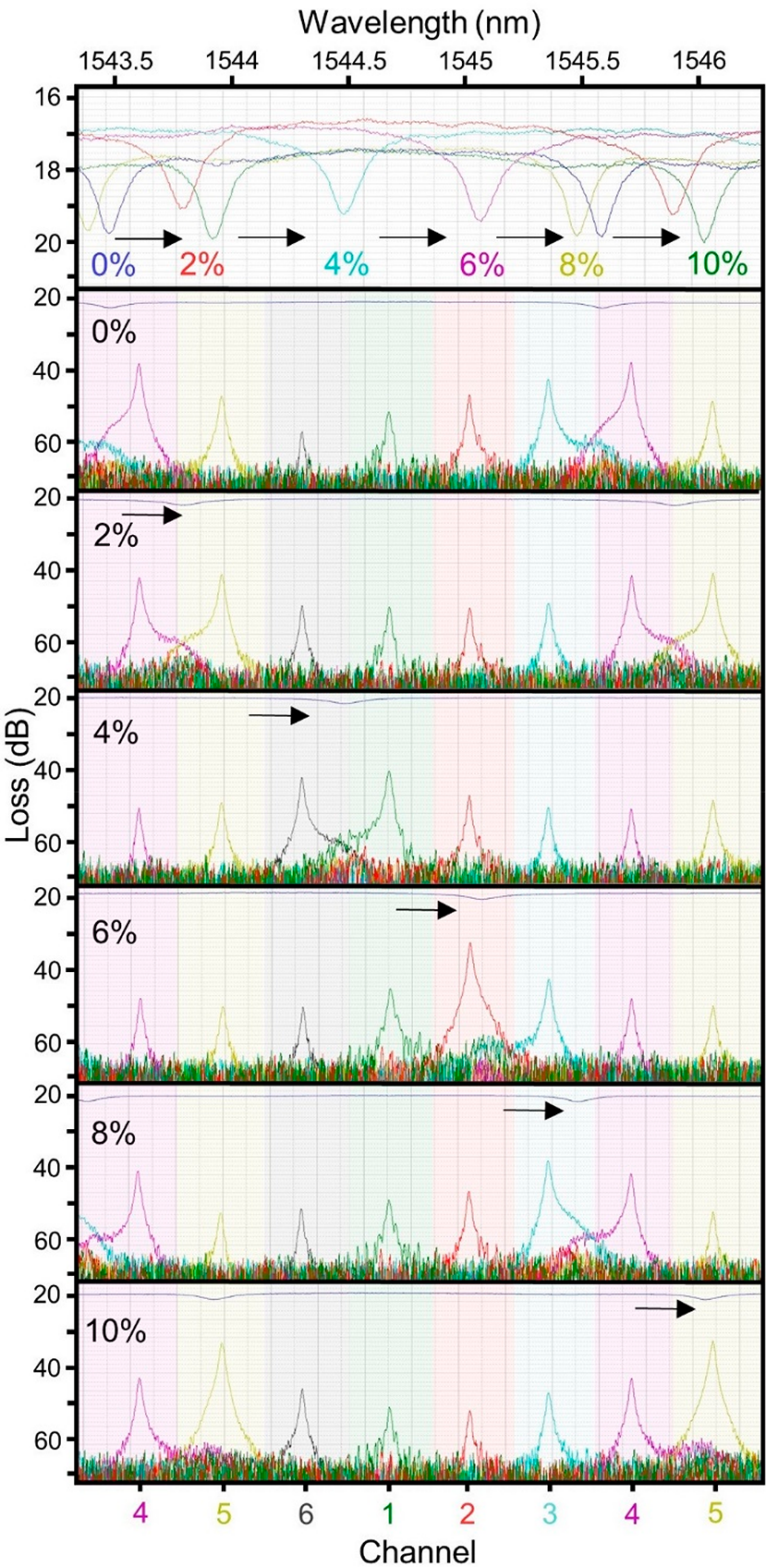
Bulk sensing sucrose solutions. Sucrose solutions spanning 0% through 10% demonstrate the passage of the sensing ring over more than one free-spectral range (FSR) of the spectrometer. The top panel isolates the transmitted spectra through the sensing ring for each sucrose solution, shown as different colors for clarity. Below, the spectrometer response is shown for each sucrose solution individually. There are six spectrometer channels, magenta, yellow, gray, green, red, and cyan. After a single FSR, the channels repeat in order. Here, each plot spans ∼3 nm, and the magenta and yellow channels are shown twice. The blue notch resonance belongs to the transmitted power T_1_ from the sensing ring which is exposed to the sucrose solutions.

To characterize the bulk sensitivity of unfunctionalized devices, several different concentrations of sucrose solutions were pumped sequentially over the surface of a single-sensing ring spectrometer. Since the refractive indices of these solutions are well-known, the refractive index sensitivity of the device can be reported in terms of shift per refractive index unit (nm/RIU). Typical spectra from a bulk sensing experiment are shown in Figure 5. When the concentration of sucrose increases from 0 to 10%, the resonance shifts over 2.4 nm, exceeding the FSR of the sensing and spectrometer rings. The average sensitivity of 152 devices measured from the redshift of the T_1_ resonance was 179.7 ± 36 nm/RIU.

At intermediate sucrose concentrations over this range, the intensity measured at each spectrometer channel is observed to increase with proximity to the resonance wavelength of the sensing ring. Whereas resolution in spectrometers with narrow channel spacing is optimized by decreasing interchannel crosstalk, in a spectrometer with sparse channel spacing and a broad source that spans several adjacent channels, identification of the central wavelength is facilitated by assessing the relative power transmitted to adjacent channels.

### Controlled Biosensing of C-Reactive Protein (CRP)

**Figure 6:**
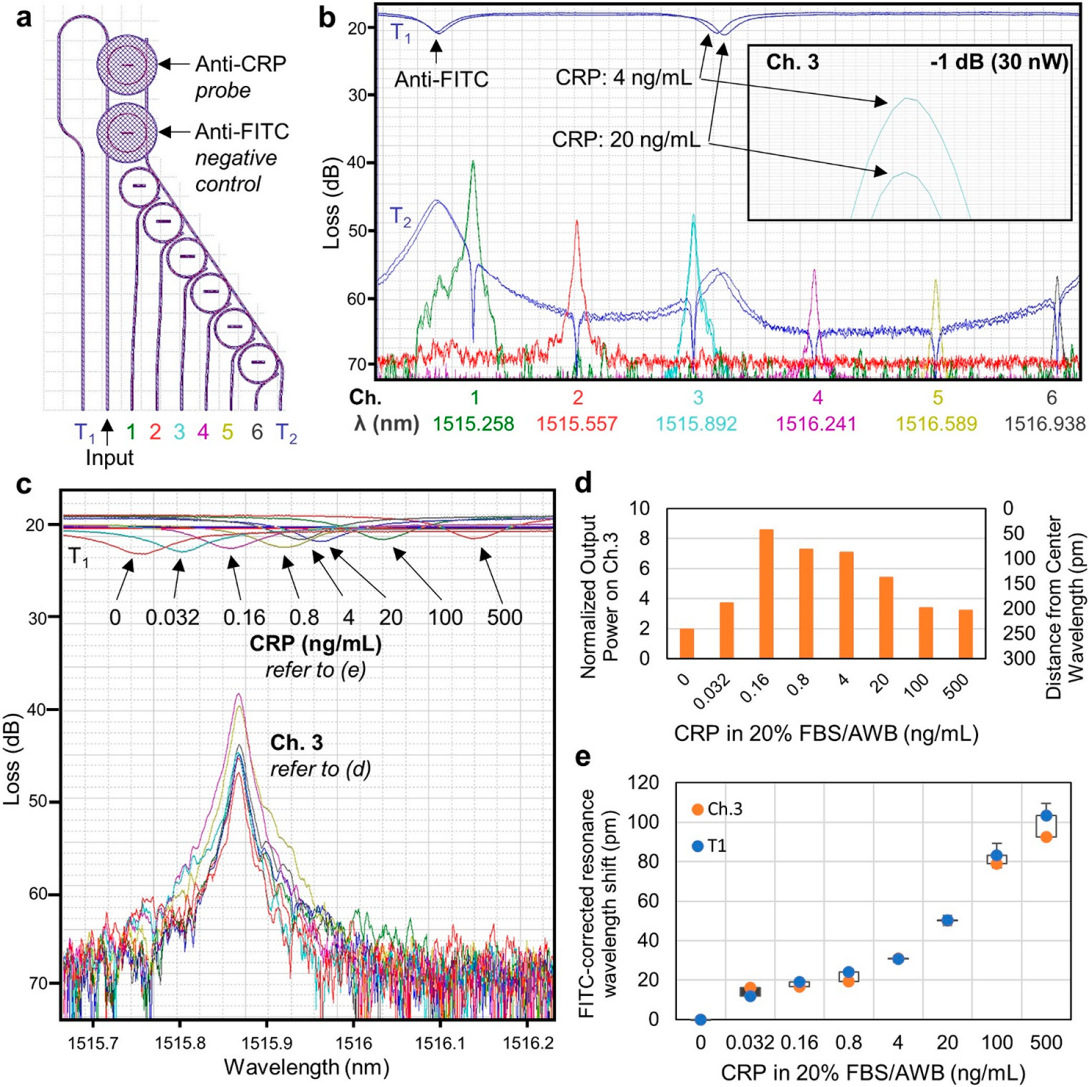
(a) Layout of the control-referenced integrated microring resonators and microring filter bank spectrometer. Input light is coupled into the second waveguide of the array. This serves as the bus waveguide for the add/drop sensing rings. Trenches are etched through the top oxide cladding to expose the nitride waveguides of the sensing (probe, anti-CRP; control, anti-FITC) rings. The transmission spectrum T_1_ is collected from the first waveguide in the array. Light dropped from the sensing rings proceeds to the array of six oxide-buried add/drop spectrometer rings, and their output is measured at the corresponding drop waveguides. Finally, the transmitted light is measured from the last waveguide in the array. (b) Superimposed sensing spectra for two different concentrations of CRP. The resonance wavelength of each sequential spectrometer peak is separated by ∼300 pm, so that the six rings uniformly span the ∼2.2 nm free spectral range (FSR) of the sensing rings. The left-most resonance notches in the transmission spectra T_1_ belong to the anti-FITC-labeled negative control ring. There is negligible shift observed for increasing concentrations of CRP. The central peaks in the transmission spectra T_1_ belong to the anti-CRP-labeled probe ring. Inset: enlargement of the third spectrometer peak from panel b. When the concentration of CRP in solution increases from 4 ng/mL to 20 ng/mL, the observed redshift leads to a decrease in the measured output power in the third spectrometer channel of 30 nW. (c) Superimposed spectra for increasing concentrations of CRP in 20% FBS in AWB. The resonance notch of the anti-CRP ring redshifts with increasing concentration of CRP in solution. (d) The power measured on the third spectrometer ring increases and then decreases as the sensing ring moves in and out of resonance with the spectrometer ring. The power is inversely proportional to the distance between the resonance wavelength of the sensing ring and the center wavelength of the spectrometer ring. (e) Dose–response curve for the FITC-corrected resonance wavelength shift (FITC-resonance shift subtracted from CRP-resonance shift to control for nonspecific interactions) with increasing concentration of CRP (*N* = 4), as measured from spectrometer channel 3 and the transmitted resonance T_1_.

CRP is a serum cytokine that has been indicated as a biomarker of inflammation and disease. FDA-approved clinical assays for CRP are routinely used.^20^ Normal levels of CRP in healthy individuals are <3 ng/mL, with values rising to more than 500 ng/mL in acute inflammation.^21^ As such, it is an ideal analyte for testing these devices in the surface-sensing regime with a clinically relevant biomarker. The layout of a dual-ring sensor integrated with a filter bank spectrometer is presented in Figure 6a. Of the nine waveguides at the bottom of the device, the second serves as the input and is coupled to two sensing rings as a bus waveguide. Transmitted light then returns to the first waveguide T_1_. Light that couples through one of the sensing rings is dropped to a second bus waveguide with six spectrometer rings buried in the oxide cladding. The resonance wavelengths of these rings are designed to be separated by 300 pm to uniformly span their free spectral range of ∼2.2 nm. As fabricated, the average separation for this device was 336 pm with a standard deviation of 19 pm. Light that is resonant with a spectrometer ring drops to its corresponding output waveguide, labeled 1 through 6. Finally, light that is transmitted beyond the filter bank spectrometer is measured from the waveguide labeled T_2_. To measure the specific binding of C-reactive protein (CRP), one sensing ring was functionalized with the anti-CRP antibody as a capture probe, while the other sensing ring was functionalized with the anti-FITC antibody as a negative control.

Typical biosensing spectra are presented in Figure 6b. Here, spectra measured for 4 ng/mL CRP are overlaid with the spectra for 20 ng/mL CRP, to show that the spectrometer can be used to resolve a 5-fold difference in concentration. The wavelength of the tunable laser source is scanned, and the output power (reported as loss (dB)) is measured for all output channels of the device. The spectrometer channels appear at the bottom of the plot uniformly distributed from green to gray. There is a negligible change in the location of the anti-FITC resonance in response to an increasing concentration of CRP, and as such there is very little change in the output power of the first spectrometer channel (the closest match to the control anti-FITC resonance). However, a readily discernible 30 nW decrease in power measured from the third spectrometer channel is observed for two sequential concentrations of CRP (Figure 6c).

Spectra for CRP diluted in 20% FBS/AWB at concentrations spanning 0–500 ng/mL are overlaid in Figure 6c. As the sensing resonance moves from left to right across the third spectrometer channel, the normalized power observed increases and subsequently decreases, as shown in Figure 6d. The observed power is inversely proportional to the separation between the resonance wavelength of the sensing ring and the center wavelength of the spectrometer ring. This relationship is used to estimate the resonance wavelength of the sensing ring from the measured output power of the spectrometer channel. With the response for both the anti-CRP and anti-FITC control ring measured on separate channels, the FITC-corrected shift is calculated by subtracting the FITC shift from the CRP shift, to correct for nonspecific binding and bulk effects. The result is the dose–response curve presented in Figure 6e. The CRP-dependent redshift in resonance wavelengths for anti-CRP and anti-FITC rings was also recorded for T_1_ for comparison with the ring filter bank spectrometer output. Differences between the T1 and spectrometer output may be attributed to error in the Lorentzian fit of resonance spectra.

### Temperature Dependence and Manufacturing Variability

**Figure 7:**
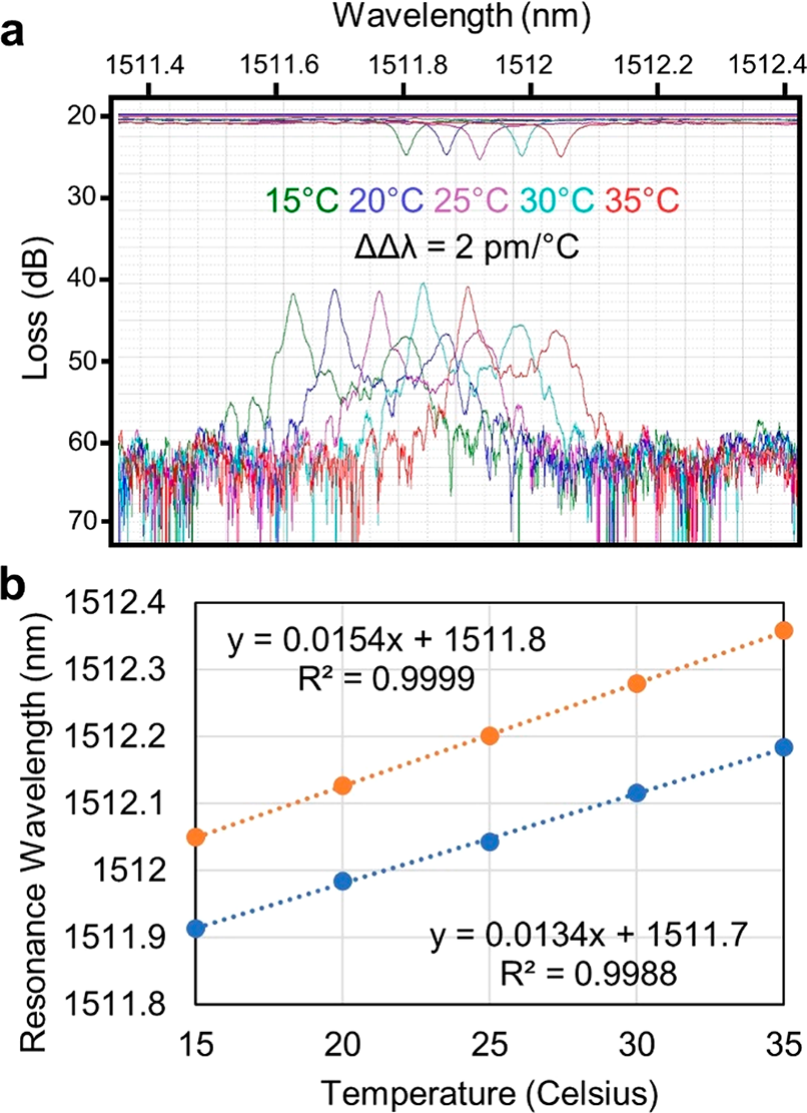
(a) Spectra of a sensing ring and the closest spectrometer channel at several temperatures spanning 15 to 35 °C. (b) Plot of the temperature dependence of the sensing and spectrometer rings. The dependence is nearly identical, deviated by only 2 pm/°C over the 20° range observed.

There are two potential concerns with regard to the use of microring filter banks for biosensing. First, since the resonant condition of microring resonators is well-known to have a temperature dependence, questions about the need for rigorous temperature control of the device might arise. This turns out to not be an issue: since the microrings used for sensing and those constituting the microring filter bank are made of the same material and are on the same chip, their sensitivity to thermal fluctuations should be quite similar if not identical. Thus, as temperature fluctuates, the resonance wavelengths of the sensors and spectrometer move together, and the observed power on a spectrometer channel is unaffected. The spectrum presented in Figure 7a demonstrates that the resonance of the sensing rings and spectrometer rings move together over a 20° range. The deviation between the two is less than 2 pm per degree Celsius (Figure 7b).

**Figure 8:**
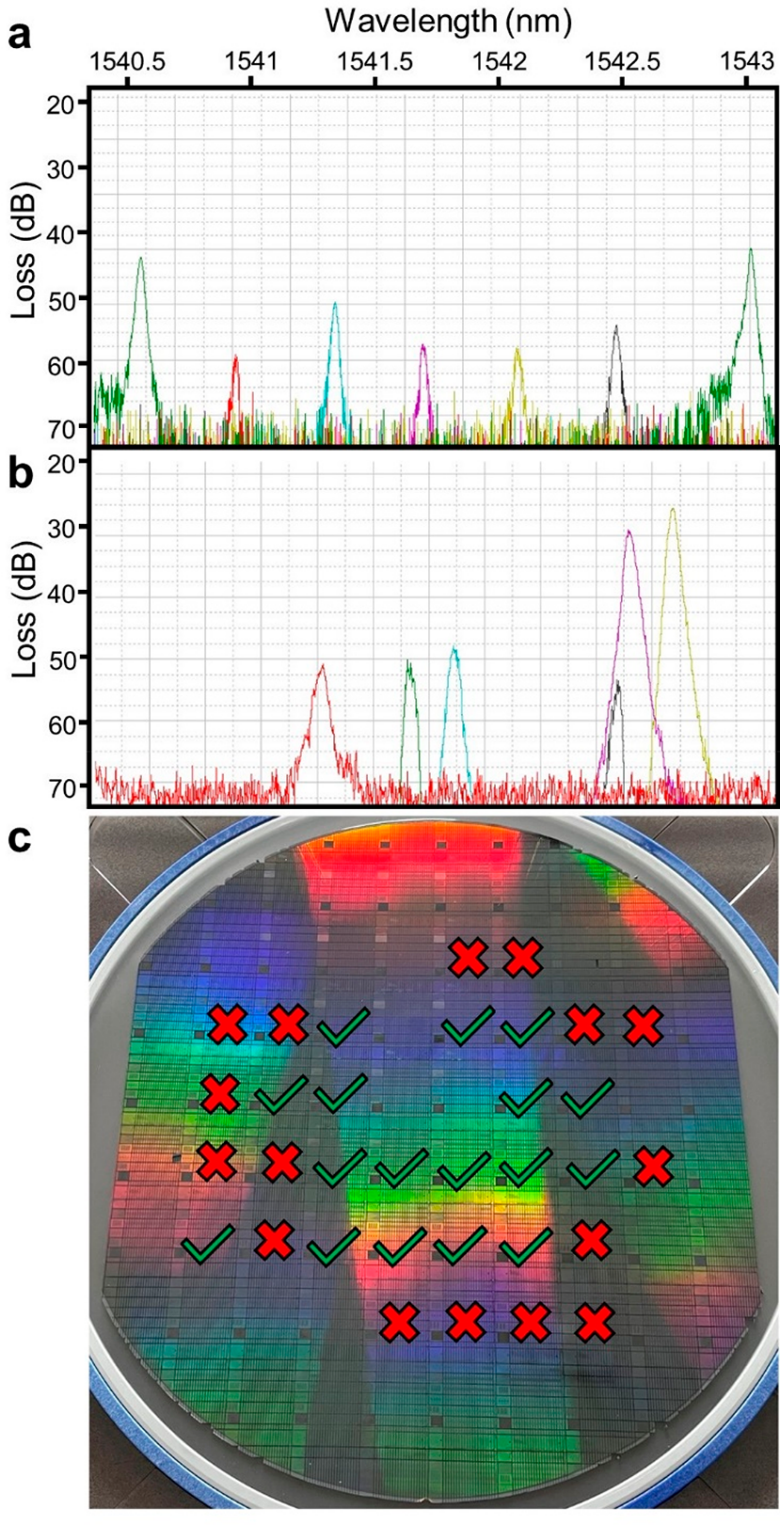
(a) The resonance wavelengths of the spectrometer rings are uniformly distributed and in the correct sequence (green, red, cyan, magenta, yellow, gray). (b) Spectra from some devices exhibited nonuniform spacing and incorrect sequence. (c) Dice with nonuniform spacing and incorrect sequence are marked with a red “X”. Dice without these flaws are marked with a green check. Of 36 dice sampled, 17 contained spacing and sequencing errors.

The second potential concern about this approach concerns the sensitivity of the design to manufacturing variability. Fabrication variations across the wafer, both stochastic and deterministic, may lead to unacceptable device properties. To test this, we acquired microring filter bank spectra from 36 devices taken from separate reticles on a single wafer. Modest variation in the refractive index of the silicon nitride may lead to dilation and contraction of the spectrometer channel spacing from one die to the next. For example, spectra presented in Figure 8a exhibit modest contraction leading to a larger gap between the final spectrometer channel (gray) and the first (green). This level of variation may be accommodated as there is not significant overlap between what should be independent channels. However, dice exhibiting spectra similar to those shown Figure 8b are unacceptable because the channel order and spacing are irregular. Of the 36 dice sampled, we judged 17 (47%) as exhibiting sufficient variation as to be unusable, and they were almost exclusively close to the perimeter of the wafer (Figure 8c).

## Discussion

In general, spectrometers are optimized for high resolution by increasing channel count and density while decreasing interchannel crosstalk. However, we have shown that a spectrometer with few, sparsely spaced channels can be used to measure the shift of an upstream sensing ring resonator. The bulk refractive index sensitivity (179.7 ± 36 nm/RIU) observed for these devices is state of the art for silicon nitride. For biosensing, CRP was detected in diluted serum at 32 pg/mL.

One of the challenges of integrating photonic sensors with spectrometers is matching the temperature dependence of each component. Here, we have shown agreement between the temperature dependence of the sensing rings and the integrated filter bank spectrometers, with a shift of less than 2 pm per degree Celsius over a 20° range. As with all photonic technologies there continues to be a need to improve fabrication yield. We anticipate that the implementation of the design for manufacturing (DFM) methodology coupled with a better understanding of wafer-scale variation in the silicon nitride refractive index will help in this regard. Additionally, since the sparse sampling approach can tolerate limited random variation of the center wavelengths of spectrometer rings, one approach to avoid overlapping channels that lead to undersampling of the sensing ring is to increase the density of spectrometer channel spacing. In this scenario, the device can be designed to tolerate one or more overlapping rings.

We also anticipate several opportunities for improving performance. In the current design of a double sensing ring-coupled filter bank, the two sensing rings are not independent. As can be seen in typical spectra, the peak intensity of the second ring is less than that of the first ring because it is downstream. This limits the ability to resolve small changes in resonance shift, because the corresponding change in spectrometer power may not be significant enough to distinguish from the noise. This issue could be resolved either by decreasing the coupling gap of the second ring to compensate for the disparity or by separating the inputs for both sensing rings. The latter approach could be accomplished by inserting a two-way splitter between the edge coupled input and the sensing rings. Of course, one could also increase confidence in the measurement via use of multiple sensor/spectrometer instances on the same chip or use multiple devices for multianalyte (multiplex) sensing. This would increase the cost of the device due to a larger chip footprint but may be warranted in some applications.

A limited amount of control can be demonstrated over setting the bandwidth of the spectrometer channel. The channel width, as measured by the full width at half maximum (fwhm), can be adjusted between ∼45 pm and ∼15 pm by increasing the coupling gap. However, a trade-off exists where increasing bandwidth decreases resolution. Varying the coupling gaps of upstream sensing rings and downstream spectrometer rings in tandem can optimize sampling of the sensing ring spectra.

There is also opportunity to split the optical signal downstream of sensing rings to address several branches of spectrometer filter rings. Such a configuration enables not only an increase of the surface density of the spectrometer but also enables an increase in ring count while decreasing the likelihood of downstream channels interfering with upstream channels in strongly coupled configurations.

Although the current footprint of a six-channel device is 1 mm × 2 mm, more efficient routing of waveguides would decrease this by half. The footprint could be decreased further by decreasing the radii of the spectrometer and sensing rings. However, this will also increase the FSR, and additional channels may be required to span the range between successive resonances.

In summary, here we have presented the integration of sensor and spectrometer microring resonators for the detection of inflammatory biomarker CRP in serum. We have shown that this configuration is tolerant to temperature variation but is also subject to fabrication variability inherent to wafer-scale photolithography. The results shown here suggest that integration of ring resonator-based biosensors with on-chip ring filter bank spectrometers represents a promising approach to inexpensive integrated sensor development.

## Supporting Information Available

Layout and photomicrographs of double sensing ring-coupled filter banks; schematic of the full measurement apparatus (PDF)

## Abbreviations

AIM: American Institute for Manufacturing
AWB: Assay Wash Buffer
AWG: Arrayed Waveguide Grating
CMT: Coupled Mode Theory
CRP: C-Reactive Protein
DFM: Design For Manufacturing
FBS: Fetal Bovine Serum
FD: Finite Difference
FITC: Fluorescein
FSR: Free-Spectral Range
fwhm: Full-Width at Half-Maximum
mPBS: Modified Phosphate-Buffered Saline
MPW: Multi-Project Wafer
PDMS: Polydimethylsiloxane
POC: Point-of-Care
RIU: Refractive Index Units

## References

[ref1] Heidt B.; Siqueira W. F.; Eersels K.; Diliën H.; van Grinsven B.; Fujiwara R. T.; Cleij T. J. Point of Care Diagnostics in Resource-Limited Settings: A Review of the Present and Future of PoC in Its Most Needed Environment. Biosensors 2020, 10 (10), 133 10.3390/bios10100133. 32987809 PMC7598644

[ref2] Kelley S. O. Challenges and Opportunities for Wearable Sensing Systems. ACS Sens. 2022, 7 (2), 345–346. 10.1021/acssensors.2c00284. 35209717

[ref3] Cognetti J. S.; Steiner D. J.; Abedin M.; Bryan M. R.; Shanahan C.; Tokranova N.; Young E.; Klose A. M.; Zavriyev A.; Judy N.; Piorek B.; Meinhart C.; Jakubowicz R.; Warren H.; Cady N. C.; Miller B. L. Disposable Photonics for Cost-Effective Clinical Bioassays: Application to COVID-19 Antibody Testing. Lab Chip 2021, 21 (15), 2913–2921. 10.1039/D1LC00369K. 34160511

[ref4] Luchansky M. S.; Washburn A. L.; McClellan M. S.; Bailey R. C. Sensitive On-Chip Detection of a Protein Biomarker in Human Serum and Plasma over an Extended Dynamic Range Using Silicon Photonic Microring Resonators and Sub-Micron Beads. Lab Chip 2011, 11 (12), 2042–2044. 10.1039/c1lc20231f. 21541438 PMC3116709

[ref5] Chakravarty S.; Lai W.-C.; Zou Y.; Drabkin H. A.; Gemmill R. M.; Simon G. R.; Chin S. H.; Chen R. T. Multiplexed Specific Label-Free Detection of NCI-H358 Lung Cancer Cell Line Lysates with Silicon Based Photonic Crystal Microcavity Biosensors. Biosens. Bioelectron. 2013, 43, 50–55. 10.1016/j.bios.2012.11.012. 23274197 PMC3594323

[ref6] Baker J. E.; Sriram R.; Miller B. L. Recognition-Mediated Particle Detection under Microfluidic Flow with Waveguide-Coupled 2D Photonic Crystals: Towards Integrated Photonic Virus Detectors. Lab Chip 2017, 17 (9), 1570–1577. 10.1039/C7LC00221A. 28357424 PMC5482269

[ref7] Baker J. E.; Sriram R.; Miller B. L. Two-Dimensional Photonic Crystals for Sensitive Microscale Chemical and Biochemical Sensing. Lab Chip 2015, 15 (4), 971–990. 10.1039/C4LC01208A. 25563402 PMC4315696

[ref8] Luff B. J.; Wilkinson J. S.; Piehler J.; Hollenbach U.; Ingenhoff J.; Fabricius N. Integrated Optical Mach-Zehnder Biosensor. Journal of Lightwave Technology 1998, 16 (4), 583–592. 10.1109/50.664067.

[ref9] Goodwin M. J.; Besselink G. A. J.; Falke F.; Everhardt A. S.; Cornelissen J. J. L. M.; Huskens J. Highly Sensitive Protein Detection by Asymmetric Mach-Zehnder Interferometry for Biosensing Applications. ACS Appl. Bio Mater. 2020, 3 (7), 4566–4572. 10.1021/acsabm.0c00491. 35025455

[ref10] Bryan M. R.; Miller B. L. Silicon Optical Sensor Arrays for Environmental and Health Applications. Current Opinion in Environmental Science & Health 2019, 10, 22–29. 10.1016/j.coesh.2019.09.005.

[ref11] Takahashi H.; Suzuki S.; Kato K.; Nishi I. Arrayed-Waveguide Grating for Wavelength Division Multi/Demultiplexer with Nanometre Resolution. Electron. Lett. 1990, 26 (2), 87–88. 10.1049/el:19900058.

[ref12] Cheng R.; Zou C.-L.; Guo X.; Wang S.; Han X.; Tang H. X. Broadband On-Chip Single-Photon Spectrometer. Nat. Commun. 2019, 10 (1), 4104 10.1038/s41467-019-12149-x. 31506440 PMC6736971

[ref13] Ma K.; Chen K.; Zhu N.; Liu L.; He S. High-Resolution Compact On-Chip Spectrometer Based on an Echelle Grating With Densely Packed Waveguide Array. IEEE Photonics Journal 2019, 11 (1), 1–7. 10.1109/JPHOT.2018.2888592.

[ref14] Subramanian A. Z.; Ryckeboer E.; Dhakal A.; Peyskens F.; Malik A.; Kuyken B.; Zhao H.; Pathak S.; Ruocco A.; De Groote A.; Wuytens P.; Martens D.; Leo F.; Xie W.; Dave U. D.; Muneeb M.; Van Dorpe P.; Van Campenhout J.; Bogaerts W.; Bienstman P.; Le Thomas N.; Van Thourhout D.; Hens Z.; Roelkens G.; Baets R. Silicon and Silicon Nitride Photonic Circuits for Spectroscopic Sensing On-a-Chip [Invited]. Photon. Res. 2015, 3 (5), B47–B59. 10.1364/PRJ.3.000B47.

[ref15] Martens D.; Ramirez-Priego P.; Murib M. S.; Elamin A. A.; Gonzalez-Guerrero A. B.; Stehr M.; Jonas F.; Anton B.; Hlawatsch N.; Soetaert P.; Vos R.; Stassen A.; Severi S.; Van Roy W.; Bockstaele R.; Becker H.; Singh M.; Lechuga L. M.; Bienstman P. A Low-Cost Integrated Biosensing Platform Based on SiN Nanophotonics for Biomarker Detection in Urine. Analytical Methods 2018, 10 (25), 3066–3073. 10.1039/C8AY00666K.

[ref16] Zhang Z.; Wang Y.; Tsang H. K. Tandem Configuration of Microrings and Arrayed Waveguide Gratings for a High-Resolution and Broadband Stationary Optical Spectrometer at 860 Nm. ACS Photonics 2021, 8 (5), 1251–1257. 10.1021/acsphotonics.0c01932.

[ref17] Holzwarth C. W.; Barwicz T.; Popović M. A.; Rakich P. T.; Ippen E. P.; Kärtner F. X.; Smith H. I. Accurate Resonant Frequency Spacing of Microring Filters without Postfabrication Trimming. Journal of Vacuum Science & Technology B: Microelectronics and Nanometer Structures Processing, Measurement, and Phenomena 2006, 24 (6), 3244–3247. 10.1116/1.2363402.

[ref18] Zhou J.; Husseini D. A.; Li J.; Lin Z.; Sukhishvili S.; Coté G. L.; Gutierrez-Osuna R.; Lin P. T. Mid-Infrared Serial Microring Resonator Array for Real-Time Detection of Vapor-Phase Volatile Organic Compounds. Anal. Chem. 2022, 94 (31), 11008–11015. 10.1021/acs.analchem.2c01463. 35912577

[ref19] Fahrenkopf N. M.; McDonough C.; Leake G. L.; Su Z.; Timurdogan E.; Coolbaugh D. D. The AIM Photonics MPW: A Highly Accessible Cutting Edge Technology for Rapid Prototyping of Photonic Integrated Circuits. IEEE J. Sel. Top. Quantum Electron. 2019, 25 (5), 1–6. 10.1109/JSTQE.2019.2935698.

[ref20] Oberhoffer M.; Rußwurm S.; Bredle D.; Chatzinicolaou K.; Reinhart K. Discriminative Power of Inflammatory Markers for Prediction of Tumor Necrosis Factor-α and Interleukin-6 in ICU Patients with Systemic Inflammatory Response Syndrome (SIRS) or Sepsis at Arbitrary Time Points. Intensive Care Med. 2000, 26 (2), S170–S174. 10.1007/s001340051138. 18470714

[ref21] Nehring S. M.; Goyal A.; Patel B. C. C Reactive Protein; StatPearls Publishing, 2022. 28722873

