# Supplementary material for: Biosensing with Silicon Nitride Microring Resonators Integrated with an On-Chip Filter Bank Spectrometer
Source: ACS Sens. 2023 Feb 14;8(2):739–47. doi: 10.1021/acssensors.2c02276 (PMC9972465; doi:10.1021/acssensors.2c02276)
Supplement: Supplementary file 1 — se2c02276_si_001.pdf [file se2c02276_si_001.pdf]

Biosensing with Silicon Nitride Microring Resonators Integrated with an On-Chip Filter Bank Spectrometer

Michael R. Bryan[a,c], Jordan N. Butt[b], Joseph Bucukovski[c], Benjamin L. Miller[a,c,d]*

[a] Department of Dermatology, University of Rochester, Rochester, NY 14627, USA
[b] Department of Chemistry, University of Rochester, Rochester, NY 14627, USA
[c] Department of Biochemistry and Biophysics, University of Rochester, Rochester, NY 14627, USA
[d] Institute of Optics, University of Rochester, Rochester, NY 14627, USA

SUPPLEMENTARY INFORMATION

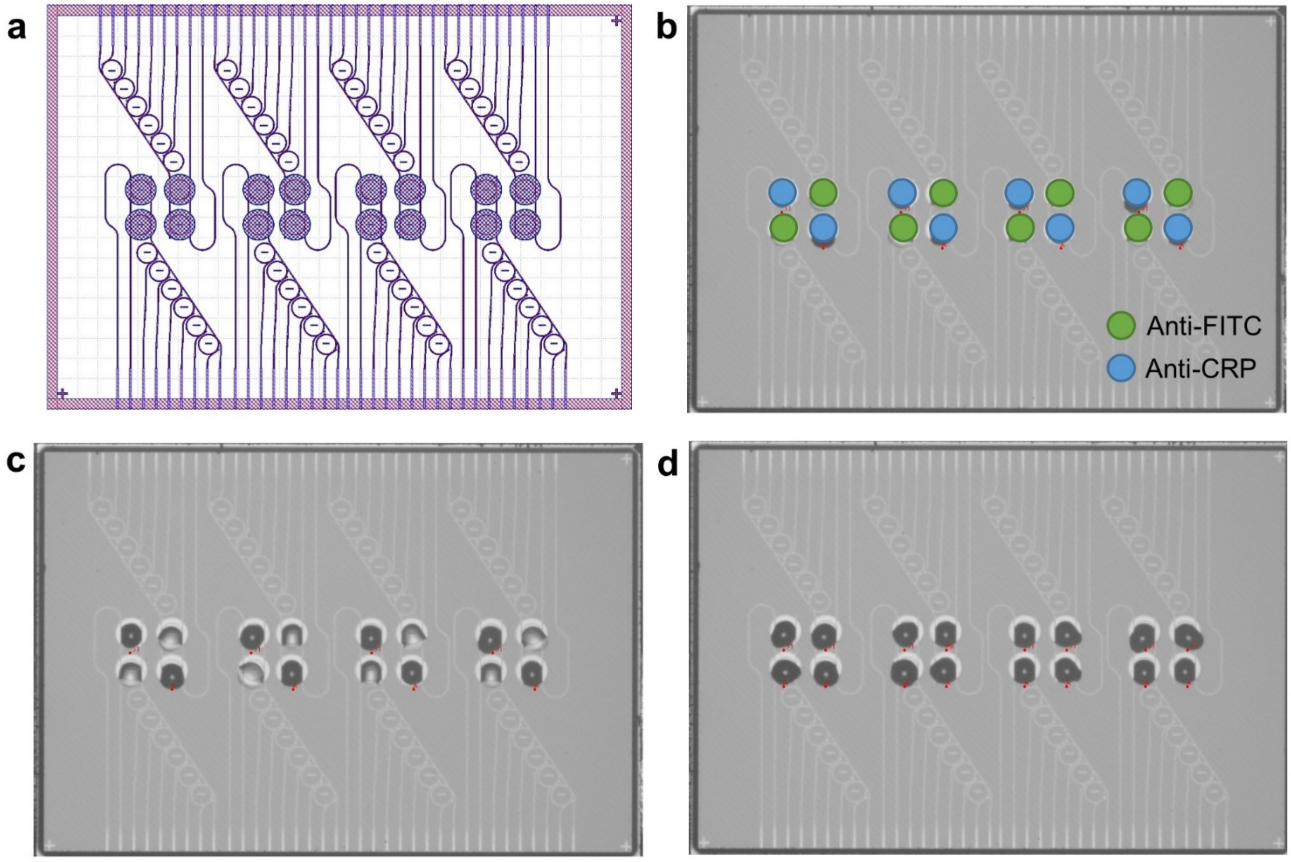

Figure S1. (a) Layout of double sensing ring-coupled filter banks (b) Location of anti-FITC and anti-CRP printed on individual sensing rings using a piezoelectric microarrayer (c) Image of antibody droplets on the sensing rings (d) Image of sensing rings after they were overspotted with StabilCoat Plus.

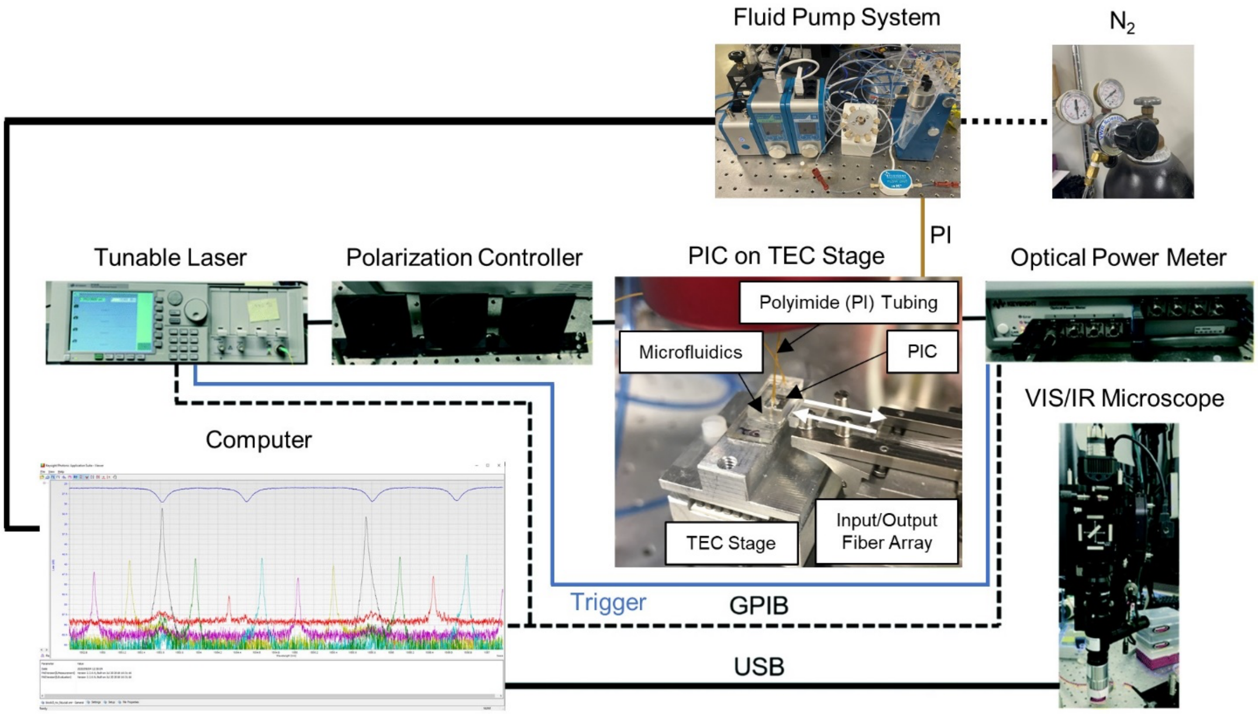

Figure S2. Schematic representation of the experimental apparatus. VIS, visible-wavelength light; IR, infrared-wavelength light; GPIB, general purpose interface bus; USB, universal serial bus.
